# Sex bias in Neolithic megalithic burials

**DOI:** 10.1002/ajpa.24645

**Published:** 2022-11-02

**Authors:** Elliot Elliott, Tina Saupe, Jess E. Thompson, John E. Robb, Christiana L. Scheib

**Affiliations:** ^1^ Estonian Biocentre, Institute of Genomics University of Tartu Tartu Estonia; ^2^ McDonald Institute for Archaeological Research University of Cambridge Cambridge UK; ^3^ Darwin College University of Cambridge Cambridge UK; ^4^ Department of Archaeology University of Cambridge Cambridge UK; ^5^ St John's College University of Cambridge Cambridge UK

**Keywords:** ancient DNA, biological sex, demography, Ireland, prehistoric archaeology, United Kingdom

## Abstract

**Objectives:**

A statistical study comparing osteological and ancient DNA determinations of sex was conducted in order to investigate whether there are sex biases in United Kingdom and Irish Neolithic megalithic burials.

**Materials and Methods:**

Genetic and osteological information from human individuals from 32 megalithic sites in the UK and Ireland dating from 4000 to 2500 cal. BCE was collected and statistically analyzed to test whether there is a true over‐representation of males at these sites. The published dataset from the study by Sánchez‐Quinto et al. in 2019 was initially analyzed before being refined and included in a larger dataset. Osteological analysis of sex bias was limited to adults with available sex estimations, and genetic analysis limited to published data

**Results:**

Two sites consistently returned significant *p*‐values suggesting a potential over‐representation in osteological males at one site (Knowe of Midhowe, Orkney) and genetic males in the other (Primrose Grange, Ireland). Cumulative statistical analyses point towards a male bias in the representation of sexes in Neolithic megalithic burials, but these results do not reflect the site‐by‐site and regional variation found in this study.

**Discussion:**

The interpretation of sex bias, that is, the over‐representation of one sex over another ‐ depends on other socio‐cultural variables (e.g., kinship) and the emphasis placed on statistical significance. The trend towards males being over‐represented in Neolithic megalithic burials is not as clear as previously thought, and requires further testing and data collection to uncover.

## 1 INTRODUCTION

In recent years, ancient DNA (aDNA) research has greatly enhanced our understanding of human demographic history. Analysis of genome‐wide data has allowed researchers to investigate the unwritten history of ancient cultures and societies. Several studies have focused on and provided insights into the movement and integration of prehistoric peoples in Western Eurasia (Brace et al., 2019; Cassidy et al., 2016; Olalde et al., 2018), as well as into paleodemographic structures (Sánchez‐Quinto et al., 2019; Fowler, 2022). In their study of genome‐wide data from selected ancient individuals from Neolithic megalithic sites in modern‐day United Kingdom, Ireland, and Sweden, Sánchez‐Quinto et al. (2019) highlighted a possible male sex‐biased admixture, that is, a bias in genetic contribution in a population, evidenced by limited Y‐chromosome haplogroup lineages and a greater diversity in mtDNA haplogroups. This was particularly evident in the samples from present‐day Scotland and to a lesser degree in the samples from present‐day Sweden, alongside an over‐representation of males in these contexts. They argued that this over‐representation of males could represent patrilineal kinship groups, as at least three individuals were related across two different Irish megalithic sites despite varying material culture within the monuments. This has significant implications for our understanding of Neolithic social structures in northwestern Europe.

While gender and sex are often intertwined, Robb and Harris (2018) have argued that gender in the European Neolithic was relational and contextually based, rather than the essentialized binary structure often interpreted as present in the Bronze Age. They point out that gender is typically interpreted as a cultural signifier of one's biological sex, and that any attempts to define gender must start from evidence of “sexable bodies,” even though this evidence may not fit the full spectrum of human gender presentation (Robb & Harris, 2018). Determining biological sex, therefore, can contribute to discussions of gender constructions in archeological contexts as one variable within the multitude of gender‐coded aspects of the burial rite (i.e. grave goods, body placement and orientation, grave construction and furnishings, etc.). Chromosomal sex generally determines secondary sex characteristics in the skeleton, but how and to what extent these characteristics are presented varies on an individual basis (for discussion on intersex individuals in archaeology, see Moilanen et al. (2021) on the Iron Age individual with possible aneuploidy XXY). Genetic sex obtained from sequencing could contribute to our knowledge and understanding of gendered structures in the Neolithic *and their dependence (or lack of) on later essentialized biological sex binaries* by providing evidence of “sexable bodies,” especially when osteological data are missing or indeterminate, and serve as a link between the physical evidence and cultural interpretation of Neolithic burial practices. In other words, utilizing combined genetic and osteological demographic data can help us answer whether or not these megalithic burials represent a possible manifestation of a male‐based kinship structure such as a patrilineal society. The methods for determining sex estimates are outlined below.

To further investigate the relationship between burial practices and cultural interpretation presented in recent research on the Neolithic, we collected genetic and osteological information from human individuals with absolute and relative radiocarbon dates from megalithic sites in the United Kingdom and Ireland dating from 4000 to 2500 cal. BCE to test whether there is a true over‐representation of males in megalithic burials or if it is a result of sample bias and/or missing data. This paper starts from the dataset published by Sánchez‐Quinto et al. (2019) in order to test if their hypothesis held true in osteological context, then adds sites with osteological and genetic sex data to curate a larger dataset for further analysis. Statistical significance analysis was then conducted to test whether the male sex‐bias hypothesis held true given more data and if there is a difference in sex representation based on osteological versus genetic data.

## 2 MATERIALS AND METHODS

Neolithic megalithic burials are defined herein as depositions of one or more human remains within above‐ground stone monuments dating to the period 4000 to 2500 cal. BCE. Megalithic burials appeared as early as 4500 cal. BCE in continental Europe, but current radiocarbon dates indicate that the phenomenon began much later in the UK and Ireland (Schulz Paulsson, 2019). These monuments range from long barrows to stone cairns, each with their own subsets and typologies (see Barrett, 1988).

**FIGURE 1:**
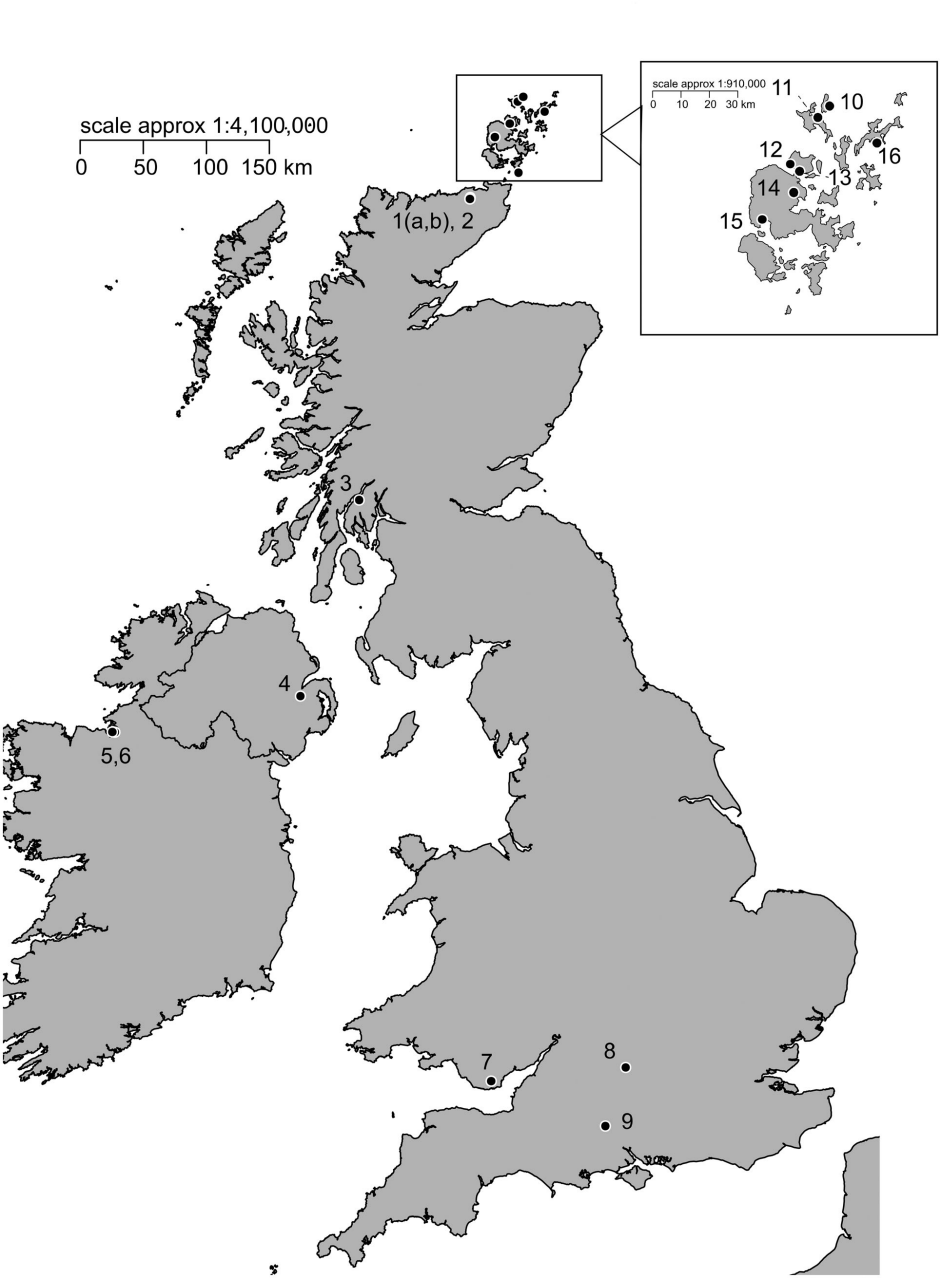
Dataset 1 sites in United Kingdom and Ireland: 1. (a) Tulloch of Assery a, Scotland (b) Tulloch of Assery B, Scotland; 2. Tulach an t'Sionnach, Scotland; 3. Clachaig, Scotland; 4. Ballynahatty, N. Ireland; 5. Carrowmore, Ireland; 6. Primrose grange, Ireland; 7. Tinkinswood, Wales; 8. Upper swell, England; 9. Fussell's lodge, England; 10. Holm of papa Westray, Orkney; 11. Point of Cott, Orkney; 12. Knowe of Lairo, Orkney; 13. Knowe of Midhowe, Orkney; 14. Isbister, Orkney; 15. Unstan, Orkney; 16. Quoyness, Orkney

Sánchez‐Quinto et al.'s study initially comprised only 24 individuals from five sites; however, we excluded the Gotland site to focus on the United Kingdom and Ireland. Their study also included individuals from Balintore, a short cist burial in Scotland; Fussell's Lodge, which, while in the United Kingdom, is a wooden structure; and the Kolin Rondel site in the Czech Republic (9470). As these two sites are, respectively, not megalithic in nature and not located in the United Kingdom and/or Ireland, they are also not included in the current analysis. Sánchez‐Quinto et al. compared their data with published genome‐wide data of 36 individuals from 17 megalithic sites and found a higher male:female ratio in megalithic burials in the British Isles than in non‐megalithic contexts (31 out of 42 samples) (*op.cit*.). The sites listed below, included in Sánchez‐Quinto et al., represent Dataset 1 in this study, comprising 36 individuals from 17 sites, with a total minimum number of individuals (MNI) of 320, 32 of which were genetically sexed and 71 of which were osteologically sexed (see Table 1, Figure 1).

**TABLE 1:** Dataset 1 sites.

Site	Location	Region
Clachaig	North Ayrshire	Scotland
Point of Cott	Orkney	Scotland
Quoyness	Orkney	Scotland
Tulach an t'Sionnach	Highland Caithness	Scotland
Tulloch of Assery A	Highland Caithness	Scotland
Tulloch of Assery B	Highland Caithness	Scotland
Knowe of Lairo	Orkney	Scotland
Knowe of Midhowe	Orkney	Scotland
Ballynahatty Compartment D	Co. Down	Ireland
Carrowmore	Sligo	Ireland
Fussell's Lodge	Wiltshire	England
Upper Swell	Chipping Norton	England
Tinkinswood	Glamorgan	Wales
Primrose Grange	Sligo	Ireland
Isbister	Orkney	Scotland
Holm of Papa Westray	Orkney	Scotland
Unstan	Orkney	Scotland

**FIGURE 2:**
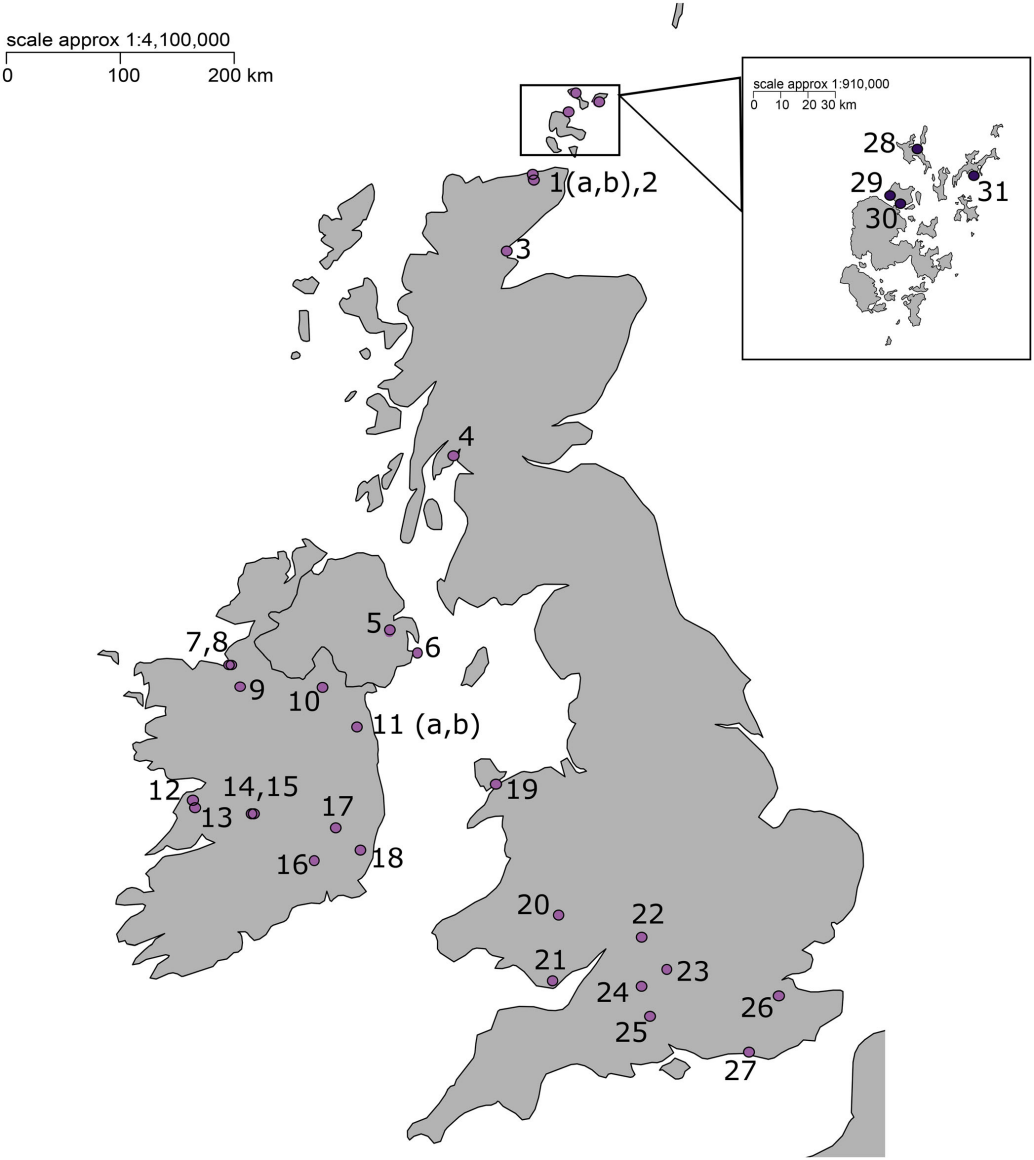
Map of Dataset 2 sites in United Kingdom and Ireland: 1. (a) Tulloch of Assery a, Scotland (b) Tulloch of Assery B, Scotland; 2. Tulach an t'Sionnach, Scotland; 3. Embo, Scotland; 4. Clachaig, Scotland; 5. Ballynahatty, N. Ireland; 6. Millin Bay, N. Ireland; 7. Carrowmore, Ireland; 8. Primrose grange, Ireland; 9. Carrowkeel, Ireland; 10. Cohaw, Ireland; 11. (a) Newgrange, Ireland (b) Newgrange site Z, Ireland; 12. Poulnabrone, Ireland; 13. Parknabinnia, Ireland; 14. Ardcroney, Ireland; 15. Ashleypark, Ireland; 16. Jerpoint, Ireland; 17. Baunogenasraid, Ireland; 18. Norrismount, Ireland; 19. Bryn Yr hen Bobl, Wales; 20. Little lodge, Wales; 21. Tinkinswood, Wales; 22. Burn ground, England; 23. Upper swell, England; 24. West Kennet, England; 25. Fussell's lodge, England; 26. Coldrum, England; 27. Point of Cott, Orkney; 28. Knowe of Lairo, Orkney; 29. Knowe of Midhowe, Orkney; 30. Quoyness, Orkney

We also compiled an expanded dataset (Dataset 2) which includes sites from Dataset 1 with sufficient published osteological sex data, as well as additional sites that have undergone recent genetic studies and which also have osteological sex data. In total, Dataset 2 comprises 32 sites with a total MNI of 442, 78 of which were genetically sexed and 149 of which were osteologically sexed (see Table 2 and Figure 2). Ultimately, analysis focused on the 78 and 149 individuals with sex data—these are not mutually exclusive, as some, but not all, of the individuals with osteological sex determinations were also genetically sexed.

**TABLE 2:** Dataset 2 sites.

Site	Location	Region
Ardcroney	Tipperary	Ireland
Ashleypark	Tipperary	Ireland
Baunogenasraid	Carlow	Ireland
Carrowkeel	Sligo	Ireland
Cohaw	Cavan	Ireland
Jerpoint	Kilkenney	Ireland
Newgrange	Co. Meath	Ireland
Newgrange Site Z	Co. Meath	Ireland
Norrismount	Wexford	Ireland
Parknabinnia	Clare	Ireland
Primrose Grange	Sligo	Ireland
Poulnabrone	Clare	Ireland
Millin Bay	Co. Down	Ireland
Ballynahatty Compartment D	Co. Down	Ireland
Carrowmore	Sligo	Ireland
Little Lodge	Powys	Wales
Bryn Yr Hen Bobl	Anglesey	Wales
Tinkinswood	Glamorgan	Wales
Coldrum	Kent	England
Fussell's Lodge	Wiltshire	England
Upper Swell	Chipping Norton	England
Burn Ground	Hampnett	England
West Kennet	Wiltshire	England
Embo	Sutherland	Scotland
Clachaig	North Ayrshire	Scotland
Point of Cott	Orkney	Scotland
Quoyness	Orkney	Scotland
Tulach an t'Sionnach	Highland Caithness	Scotland
Tulloch of Assery A	Highland Caithness	Scotland
Tulloch of Assery B	Highland Caithness	Scotland
Knowe of Lairo	Orkney	Scotland
Knowe of Midhowe	Orkney	Scotland

The group of individuals in the current analysis incorporated all suitable available data at the time of writing [2021/2022]. More individuals have been excavated from megalithic sites in the UK and Ireland, but many of these remain unsexed and/or unanalyzed. Further research establishing their sex through osteological and/or genomic analysis may allow for more far‐reaching conclusions in regards to sex demographics.

The osteological and demographic data from reports and catalogs of the 17 sites in Dataset 1 were reviewed in conjunction with the genetic data published by Sánchez‐Quinto et al. (2019). In Dataset 2, the 32 sites were analyzed in terms of osteological age and sex distribution, both individually and geographically, to check for regional patterns. Based on published reports, the remains were categorized as either “Genetic Male,” “Osteological Male,” “Osteological Possible Male,” “Genetic Female,” “Osteological Female,” “Osteological Possible Female,” “Osteological Unknown Adult,” and “Osteological Subadult” (the latter referring to individuals under approximately 18 years of age at death according to the “Updated Standards” guidelines on osteological sex assessment [Brickley & Buckberry, 2017, 33–34]).

Osteological sex is determined from sexually dimorphic skeletal traits present on the cranium, mandible, and *os coxa* (Buikstra & Ubelaker, 1994; Cox & Mays, 2000), while genetic sex is determined through extraction of aDNA usually from the petrous portion of the temporal bone or from dentine within the tooth root by calculating the ratios of X and Y chromosomes or the ratios of autosomal chromosomes (Bauer, Niederstätter, McGlynn, Stadler, & Parson, 2013, 583; Skoglund, Storå, Götherström, & Jakobsson, 2013; Fu et al., 2016; Lamnidis et al., 2018; Mittnik, Wang, Svoboda, & Krause, 2016). Both methods of sex determination rely on good preservation of specific skeletal elements, and osteological sex determination is based on phenotypical dimorphism, the expression of which has been demonstrated to vary between populations, making comparisons between modern skeletal examples of sexual dimorphism and archeological samples difficult (Inskip et al., 2019; Meindl, Lovejoy, Mensforth, & Don Carlos, 1985; Parker et al., 2020; White, Black, & Folkens, 2012). Osteological sex estimation methods are up to 98% accurate when both cranial and pelvic traits are present and well preserved (Inskip et al., 2019), but accuracy is often decreased in highly fragmented material due to differing preservation levels.

As a result of variable preservation and/or the normal variation in the skeletal presentation of secondary sex characteristics, some individuals may be given an “indeterminate” sex estimation. Both datasets in the current study include sites where the skeletal remains are often highly fragmented and commingled, and where osteological reports varied from the recent and highly detailed (such as O'Donnabhain and Tesorieri's (2014) report on Poulnabrone in Lynch, 2014) to the brief and antiquarian (such as the description of Upper Swell by Greenwell & Rolleston, 1877). A large proportion of individuals in our datasets were not assigned a sex (discussed further below), and furthermore not every “indeterminate” or “unsexed” individual underwent genetic analysis.

Furthermore, osteological sex can only be determined for individuals who have reached puberty and have sufficiently visible sexually dimorphic traits (Buikstra & Ubelaker, 1994; Cox & Mays, 2000; White et al., 2012), and therefore can only be discussed in terms of adult populations, although some novel methods have been proposed for adolescents (see Lewis, Shapland, & Watts, 2016). Therefore, in some cases, genetic sex determination was successfully undertaken on subadult remains and included in sex‐distribution analyses, but those same individuals could not be included in the analysis for the osteological data. As a result, sample size for genetic sex in some studies differs from sample size for osteological sex, which itself differs from estimated site MNI, leading to the decision in the current study to study genetic and osteological sex separately. It should be noted that previous studies, while not particularly recent, have found no correlation between sex and skeletal preservation in the archeological record, although there tends to be a degree of bias towards young adults and against subadults and elderly individuals (Bello, Thomann, Signoli, Dutour, & Andrews, 2006; Gordon & Buikstra, 1981; Stojanowski, Seidemann, & Doran, 2002; Walker, Johnson, & Lambert, 1988).

Binomial significance tests were conducted on sites with more than one genetically analyzed individual and where macroscopic analyses of age and sex of skeletal remains were undertaken and published; sites with only one genetically analyzed individual were included as part of an overall regional analysis of the sites. Four sites from Dataset 1 and nine sites from Dataset 2 ultimately had sufficient genetic and osteological data to undertake binomial testing. “Possible Male” and “Possible Female” counts were removed during a second round of analysis to test if their presence impacted statistical significance. Bonferroni multiple test corrections were then conducted in order to check for false positives.

In Dataset 1, the percentage of adults of unknown sex ranged from 40.7% in the Scotland data to 50% in the Ireland data, with the England data falling closer to the Scotland percentage at 42.1%. This inability to determine sex for a large number of individuals, either due to preservation or normal variation in sexually dimorphic traits, is a substantial limitation that should be taken into account, as this represents missing data that could significantly impact any conclusions drawn. Additionally, sites in Scotland and Ireland had more genetic samples than England in both datasets, which further complicates the statistical interpretations.

## 3 RESULTS

Given the null hypothesis that there is an equal representation of sexes in Neolithic megalithic burials in the United Kingdom and Ireland, we used the standard, but arbitrary, cut‐off value of *p* < 0.05 for determining significance.

Only three of the 17 sites in Dataset 1 had both sufficient osteological and genome‐wide data to determine p‐values based on the null hypothesis (see Table 3). Of those, the Knowe of Midhowe (Orkney) had a significance value for osteological sex (*p* = 0.011) that would suggest sex bias, but not for genetic sex (*p* = 0.25). Conversely, Primrose Grange (cf. Sánchez‐Quinto et al. 2019) had a significance value for genetic sex that suggests a bias towards males (*p* = 0.0327), but not for osteological sex. Primrose Grange was the only site of the four that required the addition of possible males and females to the overall osteological count, as they constituted half of the sexed adult population (4/8, ratio 1:2). None of the three sites had significance values for both osteological and genetic sex that would allow us to reject the null hypothesis.

**TABLE 3:** Dataset 1 sites able to undergo binomial *p*‐value analysis

Site	Location	MNI	Genetic males/total sample	Osteological males/total sexed adults	*p*‐Value genetic sex	*p*‐Value osteological sex
Knowe of Midhowe	Orkney, Scotland	25	2/2	9/10	0.25	0.011*
Quoyness	Orkney, Scotland	13	2/2	2/5	0.25	0.8125
Primrose Grange	Co. Sligo, Ireland	40	9/11	5/8	0.0327*	0.363

*Note*: Significant values notated with an asterisk (*).

The above table (Table 4) presents the results for the regional analysis of Dataset 1. Geographically, only Scotland had a significance level that would suggest a possible sex bias in both the genetic and the osteological data (genetic sex *p* = 0.0377; osteological sex including possible males *p* = 0.044). Ireland did have a significance level suggesting a possible bias in genetic sex, but that same bias is not found in the osteological data; this result may point to some unintentional sampling bias. Proportionally, genetic males did comprise a majority of the genetic samples across all three regions, ranging between 66.67% (England) and 66.9% (Ireland). The osteological proportions for males compared to total sexed adults, including possible males and females, were slightly more variable, ranging from 50% (England) to 65.7% (Scotland). When possible males and females were removed from the overall sexed adult count, this percentage increased in the Scotland data (68.2%) and the England data (52.6%), but decreased in the Ireland data from 57.1% to 50%. This variability highlights the impact of missing data points, particularly in the osteological sex data, on statistical conclusions of significance in regards to the representation of sex in these burials.

**TABLE 4:** Regional analysis of Dataset 1 sites through binomial *p*‐values and sex ratios; significant values notated with an asterisk (*)

Region	Genetic sample/osteological adults (sexed)	*p*‐Value genetic sex	*p*‐Value osteological sex (male only)	*p*‐Value osteological sex (male and possible male)	Percent genetic male (%)	Percent osteological male and possible male (%)	Percent osteological male (%)
Scotland	26/35	0.0145*	0.067	0.045*	73.1	65.7	68.2
Ireland	13/14	0.0461*	0.656	0.395	69.2	57.1	50.0
England	3/22	0.5	0.5	0.584	66.6	50.0	52.6
Total	42/71	0.00144*	0.121	0.0769	73.8	59.2	59.6

Nine of the 32 sites in Dataset 2 had sufficient osteological and genetic data for binomial testing (see Table 5). None of the nine sites had significant *p*‐values for *both* osteological and genetic sex. Interestingly, the two sites that did have significant p‐values were also included in Dataset 1: Primrose Grange, and the Knowe of Midhowe.

**TABLE 5:** Dataset 2 sites able to undergo binomial *p*‐value analyses; sites in italics were included in Dataset 1

Site	MNI	Genetic males/Total sample	Osteological males/total sexed adults	*p*‐Value genetic sex	*p*‐Value osteological sex
Carrowkeel	29	4/8	11/15	0.637	0.059
Newgrange Site Z	6	1/2	4/4	0.999	0.0625
Parknabinnia	20	8/11	2/6	0.133	0.891
*Primrose Grange*	*40*	*9/11*	*5/8*	0.0327*	*0.363*
Poulnabrone	36	8/10	4/8	0.055	0.637
Millin Bay	16	1/2	4/5	0.75	0.1875
*Point of Cott*	*13*	*1/2*	*2/3*	*0.75*	*0.5*
*Quoyness*	*13*	*2/2*	*2/5*	*0.25*	*0.8125*
*Knowe of Midhowe*	*25*	*2/2*	*9/10*	*0.25*	0.011*

*Note*: Significant values notated with an asterisk (*).

Four of the 32 sites were either single or double burials, and will be discussed separately, as they were not used for binomial testing but may be socially significant as a representation of a specific burial rite.

Table 6 presents the results for the regional analysis of Dataset 2. Overall, Ireland was the only geographic region with a significant *p*‐value for both genetic sex (*p* = 0.00000269) and osteological sex (male only *p* = 0.0394, male and possible male *p* = 0.004), indicating a bias in favor of the deposition of male adults in megalithic monuments in that region. Scotland had a significant p‐value for osteological sex, but only when possible males were included (*p* = 0.0436). The percentages of genetic males were slightly more variable than in Dataset 1, ranging from 50.0% (England) to 66.7% (Ireland). This variability is likely due to the disparity in the availability of genetic data between regions. Ireland, it seems, has more sites that have had genetic testing undertaken than England (15 sites in Ireland compared to five in England), and thus represents a project‐based bias in the data.

**TABLE 6:** Regional analysis of Dataset 2 sites through binomial *p*‐values and sex ratios; significant values notated with an asterisk (*)

Region	Genetic sample/osteological adults (sexed)	*p*‐Value genetic sex	*p*‐Value osteological sex (male only)	*p*‐Value osteological sex (male and possible male)	Percent genetic male (%)	Percent osteological male and possible male (%)	Percent osteological male (%)
Scotland	13/28	0.291	0.105	0.0436*	61.5	67.9	65.2
Ireland	56/59	0.000003*	0.0394*	0.004*	63.8	67.8	63.8
England	6/51	0.109	0.557	0.610	50.0	49.0	50.0
Wales	3/11	0.5	0.254	0.274	66.7	63.6	66.7
Total	78/149						

The osteological male percentages were less variable, both with and without the addition of possible males, although England continued to have the lowest percentage (49.0% with possible males, 50% without). It should be noted that this might be due to sampling bias error, as Wales only had three genetic samples and 11 osteologically sexed adult samples, while England had 51 osteologically sexed adult samples but only six genetic samples.

When the data from all 32 sites were combined, 91/149 (61.1%, *p* = 0.00427) adult individuals were osteologically determined to be males or possible males (see Table 7). When possible males and females were excluded, the total decreased to 75/127 (59.1%, *p* = 0.00427). Out of the 78 individuals with genetic sex data, 60 were male (76.9%, *p* = 0.00000099). It appears, therefore, that the null hypothesis cannot be rejected *unless* all sites are collated ‐ only then can an overall possible male sex bias be proposed, based on significance testing. Therefore, there is a degree of regional and site‐based variation, either due to sample and/or testing bias or to genuine differences in prehistoric cultural practices, which needs to be explored further.

**TABLE 7:** Dataset 2 total *p*‐values and sex ratios; significant values notated with an asterisk (*)

	*p*‐Value genetic sex	*p*‐Value osteological sex (male only)	*p*‐Value osteological sex (male and possible male)	Percent genetic male (%)	Percent osteological male and possible male (%)	Percent osteological male (%)
Total	0.00000099*	0.0252*	0.00427*	76.9	59.1	61.1

## 4 DISCUSSION

The statistical evidence does suggest that males tended to be buried in megalithic structures during the Neolithic period in the United Kingdom and Ireland, as originally theorized by Sanchez‐Quinto et al. However, such a generalization obscures substantial variation within the data. This is not to mention the relatively small dataset currently available for analysis: the genetic sex data represents 17.6% (78/442) and the osteological sex data 33.7% (149/442) of the estimated total MNI from the 32 sites in Dataset 2. For Dataset 1, Scotland had statistically significant evidence for a bias in male representation, whereas in Dataset 2, Ireland had more evidence for the same bias. This may be due to the fact that in Dataset 1, samples from Scotland represented 61.9% of the genetic data (26/42) and 49.2% of the osteological data (35/71), while in Dataset 2, Ireland made up a larger portion of the genetic and osteological data (71.8% and 39.6%, respectively). More data, therefore, suggests more sex bias.

Of the sites able to undergo binomial analyses from both Dataset 1 and 2, only two sites produced *p*‐values that would indicate statistical significance in biased sex representation ‐ one from Ireland (Primrose Grange), and one from Scotland (Knowe of Midhowe). Primrose Grange has evidence for genetic sex bias, which becomes interesting when juxtaposing the 9 genetic males out of 11 at the site with its osteological data, where out of a site MNI of 40, only five are estimated to be male. It is a difficult site to analyze, in part because one of the two cists that it constitutes has not undergone further osteological analysis and because it is a commingled burial, which in any situation complicates element/individual representation. In other words, Primrose Grange exemplifies the problem of missing data; about a quarter of the individuals have undergone genome‐wide analysis, only half have undergone osteological analysis, and only a portion of that half are adults able to be given a sex estimation. The Knowe of Midhowe is similarly complicated, with a MNI of 25 but only two genetic samples and 10 osteological adults with sex estimations. It is not certain, however, whether these sites can be interpreted as having an over‐representation of males based on the statistical evaluation of the available data (see the note on statistics and *p*‐values below).

Out of the 32 sites in Dataset 2, 15 had more than 50% osteological male adults represented, including four sites with single or double burials that will be discussed below. However, approximately 36% (106/294) of the total adult skeletal remains in Dataset 2 could not be assigned an osteological sex determination, and there is currently insufficient genetic data to make up this disparity. In order to increase the amount of known data, therefore, more aDNA testing on previously untested and tested sites should be undertaken, as well as a re‐evaluation of the skeletal remains from these sites, especially those that are commingled. At present, there simply is not enough data to make any firm interpretations regarding sex representation at these sites. There is no trend for male sex bias, as the results differ depending on the dataset and the region analyzed, but there *is* a tendency for sex‐specific bias in the male direction that needs to be investigated further.

This conclusion contrasts the recently published study on Hazleton North, England (Fowler et al., 2022). From an estimated 41 MNI at the site, approximately 22 of which are adults, genome‐wide data was generated for 35 individuals. Surprisingly, 27 of these individuals formed a “virilocal patrilineal descent system” (Fowler, 2022, p. 7), meaning that the individuals were descended from a single male ancestor (NC1M) and the four women with whom he reproduced. The four women may have determined their kin's placement within the chambered tomb (Fowler, 2022; Fowler et al., 2022). The study is an exceptional example of what we should be striving for, as the genetic data for almost an entire site allows for a full contextualization of the archeological data that could previously not be carried out.

## 5 KINSHIP

Patrilineal kinship, where lineage based on male descent is prioritized, has been argued to be a defining parameter of the organization of Neolithic megalithic burials (Cassidy et al., 2020; Fowler et al., 2022). This has been evidenced through genetic relationships ‐ both distant and close—based on shared Y‐chromosome and mitochondrial haplotypes and kinship coefficients between individuals interred in major megalithic sites such as Hazleton North, Carrowmore, Primrose Grange, Carrowkeel, and Newgrange (Cassidy et al., 2020; Fowler et al., 2022; Sánchez‐Quinto et al., 2019). For Hazleton North, where 23 out of 25 males shared the Y‐chromosome haplogroup I2a1b1a1a1, it appears that patrilineal descent inferred from Y‐chromosome haplotype and relationship to a particular individual determined who was buried in the tomb; but maternal sublineage based on mitochondrial DNA determined where *within* the tomb the individual was placed (Fowler et al., 2022). Cassidy et al. (2020) found that their sample of Poulnabrone and Parknabinnia individuals were significantly different in terms of Y‐chromosome haplogroup representation, with the latter representing predominantly I2a1a1b lineages (5 out of 8 individuals) and the former I2a1b1a1 lineages (8 out of 10 individuals). They argued that this, coupled with the lack of close kin within the tombs, indicated broad social differentiation that emphasized patrilineal descent. Considering that Parknabinnia spans over 1000 years and Poulnabrone 600 years, it is perhaps no surprise that no close kin were found amongst the individuals sampled from these tombs. The difference in Y‐chromosome haplogroup lineages may, on the other hand, have more to do with patrilocality, that is, lack of influx or migration of male individuals, than with patrilinity. For example, if the data and structure observed from Hazleton North are taken as a model for patrilinity in megalithic burials, then more individuals within these types of tombs should share Y‐chromosome haplogroups.

In the two sites with statistical sex bias in the current study, Primrose Grange has perhaps the most evidence for kinship, but that is only for five individuals ‐ a father/daughter pair (Primrose 2 and 17) and a second‐degree relationship between Primrose 17 and 18 and between Primrose 6 and 7, both of whom are also second‐degree related to a male individual at Carrowmore (Carrowmore 4) (Cassidy et al., 2020; Fowler et al., 2022; Sánchez‐Quinto et al., 2019, 9472). The Carrowmore individual, in turn, is distantly related to individuals at Newgrange (NG10, male), Carrowkeel (CAK533, female; CAK532, male; CAK530, female), and Millin Bay (MB6, male), which together form a distinct clade within passage tomb burials in Ireland, suggesting an identity‐by‐descent social structure based on biological relationships (Cassidy et al., 2020). This connection between megalithic burials—Primrose Grange, Carrowmore, Newgrange, Carrowkeel, and Millin Bay—albeit through increasingly distant relationships, could hint at a particular family line connected to a single male ancestor, Carrowmore 4. One family line does not, however, provide enough evidence of widespread patrilineal structure that shaped all Neolithic societies across the United Kingdom and Ireland.

Perhaps evidence of a social emphasis on males and male lineages can be found in burials that focus on a sole individual. Single and double burials appear to be exclusively part of a megalithic burial type specific to south‐eastern Ireland between 3700 and 3200 cal BCE known as Linkardstown‐type cists. Diagnostically, these burials include the deposition of a single individual or small number of individuals in a central stone cist along with a specific set of grave goods including decorated bipartite bowls (Cassidy et al., 2020, p. 24). Cassidy et al. state that there are fewer than 15 of these types of burials (ibid), and of those, four are included in Dataset 2. Since Linkardstown‐type cists are a minority region‐specific burial rite, they should be seen as outliers within the megalithic‐type burials data set. Interestingly, two individuals, those buried at Jerpoint (JP14) and Baunogenasraid (BG72), were found by Cassidy et al. to share a rare Y‐chromosome haplogroup (H2a) (Cassidy et al., 2020, SI 27). This does not necessarily indicate patrilineality, but does suggest, along with the predominance of males in the four Linkardstown‐type cists in Dataset 2, that this might be a rite that emphasizes the importance of certain male individuals who were buried in certain types of tombs. Further data, however, is required.

A potential example of a similar rite in England is the site of Trumpington Meadows, Cambridgeshire, which was not included in the present study due to typological reasons, as it is technically not a megalith. Two of the three male individuals buried in one of the earth and timber Long Barrows at the site were determined to be brothers who died several years apart, and thus may represent the specific selection of male kin to be buried in a socially significant monument (Scheib et al., 2019; Scheib, personal communication, 2022). This interpretation should be taken with caution, however, since the remains found in the contemporary adjacent monument could not be analyzed osteologically (Evans et al., 2019), which results in a lack of contextual data.

Many of these interpretations of genetic kinship are predicated on the idea of a monolithic megalith culture, itself an oversimplification of prehistoric cultures that should be taken with caution. It is entirely plausible that some cultural groups used megalithic funerary structures to bury and/or commemorate specific familial groups or lineages, or entire communities with varying kinship relationships, while others used them for single or pairs of individuals. These structures span 2500 years across a large geographical range with a great degree of morphological differences; variation in burial rite and usage, even between contemporaries, is to be expected, and the relationship between the structures and the people(s) that used them to be dynamic and negotiable. This is not to invalidate interpretations and arguments for patrilineality or patrilocality or even genetic relationships between and within megaliths, but rather to put these arguments into archeological context. There is, therefore, another variable to consider in the analysis of these megalithic burials: time.

## 6 TEMPORAL VARIATION

Not only is there a great variability in the periods these structures were in use, but there is also a large variation in the estimated length of time they were used for. Fussell's Lodge, for example, was only in use for 95 years, whereas Norrismount is estimated to have been in use for 1500 years. On average, however, the mean amount of time the selected megaliths were in use is around 500 years (505 years including Fussell's Lodge and Norrismount, 482 years excluding those outliers), and were most often in use for around 200 years.

Some sites are given generalized time frames based on tomb morphology, stratigraphic evidence, and grave goods in the absence of radiocarbon dating. Others, such as Fussell's Lodge and West Kennet, have undergone thorough radiocarbon dating and analysis (see: Wysocki, Bayliss & Whittle 2007a, 2007b). The megalith burials in modern‐day Ireland, here including Northern Ireland for geographical purposes, have undergone thorough dating programs through both morphological and radiocarbon dating analysis. The results of these programs suggest that certain tomb types were restricted to the Early Neolithic, such as the portal tombs, while others were more long lived, such as the court tombs, some of which were in use until the first half of the third millennium BCE (Cassidy et al. 2020 SI: 7–24).

Such variation in time scale and usage, in conjunction with tomb morphology and the range of burial practices present within these megaliths, indicates that these burial structures should not be seen as a monolithic whole representing a single culture or population, but rather as the result of similar traditions specific to a number of local cultures that grew and evolved over time.

## 7 A NOTE ON STATISTICS AND P‐VALUES

As Valeggia and Fernández‐Duque (2022) have noted, biological anthropology is often characterized by small sample sizes, as illustrated here, and perhaps conclusions should not be drawn based on whether an effect was found to be statistically significant. They argued that, “There is no substitute for a sound analytical framework […] followed by an interpretation of results in context,” and that “a result could be biologically, or socially important, [but] still fail to reach statistical significance” (Valeggia & Fernández‐Duque, 2022, 194). While the overall statistics based on all the available data suggest a significant bias towards the representation of males in Neolithic megalithic burials, it depends entirely on the dataset chosen and the specific region analyzed. When Bonferroni multiple test corrections were applied, however, none of the individual site p‐value results were found to be significant. The regional p‐values varied when the multiple test corrections were applied, with an overall trend towards not significant results. On a site‐by‐site and even regional basis, there is a degree of variation that cannot be overlooked and may hint at either a larger societal explanation or a more familial reflection (see the discussion of Hazleton North by Fowler et al., 2022). The question then becomes how we can further add to the available data in order to build a larger dataset that could better fit statistical parameters of significance testing.

## 8 CONCLUSION

It has been argued that evidence of a bias towards the burial of males in Neolithic megalithic tombs in northern Europe supports a patrilineal system of social organization (Cassidy et al., 2020; Fowler et al., 2022; Sánchez‐Quinto et al., 2019). In this paper, we assembled an enlarged database and subjected it to statistical significance testing on an individual, regional, and combined basis, considering both skeletally sexed data and genetically sexed data. Our findings make the argument of patrilineality, or at least a male‐dominated society, less certain while not entirely rejecting it. Regionally, outside of the Irish data, there does not appear to be a statistically significant bias in male representation in the sites in Dataset 2, according to binomial p‐values. The Irish megalith tradition may represent a specific, local male‐centered social structure, one where sex and/or gender along with social status and kinship influences the type of tomb and the funerary rites and traditions that surround it.

The overall percentage of males, genetic and osteological, does appear to outweigh the percentage of females, although these numbers should be viewed with a degree of caution due to missing data, in the form of unsexed and subadult individuals in the osteological data, and small sample sizes in the genetic data. This does not mean that there is no male sex bias, but rather that it cannot be proven given the variable nature of the osteological data as a result of preservation or excavation conditions and the currently small number of genetic samples.

Further research is therefore needed to determine whether there is an actual sex bias present in these and other contemporary burials. Osteological collections need to be revisited and a more efficient, low cost option for genetic analysis pursued so that more data can be collected from archeological remains. To echo a sentiment expressed in Sánchez‐Quinto et al.'s Supplement to their 2019 article, only when there is a large enough sample size of both genetic and osteological data from Neolithic megalithic burials and other funerary contexts in the UK and Ireland can the social and funerary dynamics of these cultures be fully analyzed and understood.

## AUTHOR CONTRIBUTIONS

**Elliot Elliott:** Conceptualization (lead); data curation (lead); formal analysis (lead); investigation (lead); methodology (lead); visualization (lead); writing – original draft (lead); writing – review and editing (equal). **Christiana Scheib:** Conceptualization (supporting); funding acquisition (lead); methodology (supporting); supervision (lead); writing – review and editing (supporting). **Tina Saupe:** Data curation (supporting); methodology (supporting); writing – review and editing (supporting). **Jess Emma Thompson:** Investigation (supporting); writing – review and editing (supporting). **John E. Robb:** Writing – review and editing (supporting).

## CONFLICT OF INTEREST

The authors declare they have no conflict of interest.

## Supporting information


**Appendix S1:** Supplementary Data: Sex_Representation_Data

## Data Availability

The data that supports the findings of this study are available in the supplementary material of this article.
